# Impact of Substrate Material, Esthetic Material Thickness, and Cement on Color Reproduction in Implant-Supported Fixed Restorations

**DOI:** 10.7759/cureus.71227

**Published:** 2024-10-10

**Authors:** Luminita Oancea, Corina Marilena Cristache, Andrei Macris

**Affiliations:** 1 Prosthetic Dentistry, Carol Davila University of Medicine and Pharmacy, Bucharest, ROU; 2 Dental Technique, Carol Davila University of Medicine and Pharmacy, Bucharest, ROU

**Keywords:** cad-cam technology, color reproduction, esthetic materials, implant-supported restorations, substrate material

## Abstract

Purpose

This study aimed to evaluate the impact of substrate material, esthetic material type and thickness, and cement shade on the final color reproduction of implant-supported fixed restorations. The goal was to identify optimal combinations for achieving clinically acceptable esthetic outcomes.

Material and methods

An in vitro study was conducted using four substrate materials, hybrid polyetherketoneketone (PEEK)-based ceramic-reinforced polymer (BioHPP), chromium-cobalt alloy (CrCo), grade 5 titanium (Ti), and white zirconium oxide ceramic (WZirCAD), and three esthetic materials, lithium disilicate ceramic (e.max CAD), polymethyl methacrylate (PMMA), and zirconia oxide ceramic (e.max ZirCAD), at five different thicknesses (0.5, 1, 1.5, 2, and 2.5 mm). Color differences (ΔE*) were measured using a spectrophotometer, both with and without cement application. Statistical analysis was performed using the Kruskal-Wallis test and Bonferroni correction to assess the effects of material combinations on color reproduction.

Results

The study found that a 1 mm thickness of e.max CAD on BioHPP and CrCo substrates provided the best color matching, with ΔE* values closest to clinical acceptability. PMMA showed higher ΔE* values, indicating lower color stability compared to e.max CAD and e.max ZirCAD. Cement shade had a near-significant influence on final color perception, particularly with e.max ZirCAD on CrCo substrates.

Conclusions

The study suggests that using e.max CAD at 1 mm thickness on BioHPP or CrCo substrates provides superior esthetic results, underscoring the need for careful material selection in clinical practice. Further in vivo research is recommended to validate these findings and explore long-term outcomes.

## Introduction

Implant-supported fixed restorations are widely recognized as an effective solution for the replacement of one or more missing teeth, as well as for full-arch fixed dental prostheses, with demonstrated high cumulative survival rates [1]. To achieve optimal outcomes, it is critical to consider various factors including the selection of an appropriate framework material, the natural appearance of the esthetic component, the creation of a natural-looking emergence profile, the choice of optimal abutment types, and the design of the prosthesis. The selection of the prosthetic framework material is crucial for minimizing biomechanical complications and plays a significant role in achieving successful esthetics. The translucency, opacity, color, opalescence, and properties of light absorption, reflection, and transmission of the material are extremely important for enhancing the esthetic appeal of the final restoration [2].

Fixed implant prostheses can be fabricated using either computer-aided design and computer-aided manufacturing (CAD-CAM) or conventional lost-wax casting methods [3]. The CAD-CAM technique has demonstrated the ability to produce implant frameworks with superior fitting accuracy, reduced distortion, and lower susceptibility to human error compared to cast frameworks while also providing excellent durability.

Polyetherketoneketone (PEKK), chromium-cobalt alloy (CrCo), titanium (Ti), and zirconia (Zir) are widely used materials in both CAD-CAM and conventional processing of fixed prosthetic frameworks, known for their good mechanical properties, fit, and biocompatibility. PEEK is an emerging dental material characterized by its semi-crystalline, linear thermoplastic structure and favorable mechanical properties [4]. Its inherent white color and adaptability in CAD-CAM processes make PEEK highly suitable for various dental applications, including fixed and removable prostheses and orthodontic appliances [5].

CrCo, commonly used in framework manufacturing, is valued for its high wear resistance, stiffness, and corrosion resistance. However, its gray color can detract from the esthetic appeal of restorations. Advances in CAD-CAM, selective laser melting (SLM), and computer numerical control (CNC) milling have further enhanced the mechanical properties of CrCo prosthetic structures [6].

Ti is widely utilized in dental implants, abutments, as well as frameworks, due to its superior mechanical properties, biocompatibility, and durability, serving as a viable alternative to gold alloys. Nonetheless, titanium abutments may impart a metallic hue or grayish tint to surrounding soft tissues, potentially compromising esthetic outcomes [7].

Zir has become popular in dental applications due to its excellent mechanical properties and tooth-like color [8]. Zirconia-alumina composites, such as alumina-toughened zirconia and alumina-toughened zirconia nanocomposites, offer robust mechanical strength and are suitable for implant abutments and frameworks. However, these materials may not always achieve optimal esthetic results. High-translucent zirconia, including yttria-stabilized tetragonal zirconia polycrystal (3Y-TZP) and partially stabilized zirconia (5Y-PSZ), can be enhanced with internal and external staining techniques to improve color matching and translucency [9].

While the mechanical properties of the substructure in full-arch implant restorations are crucial, achieving a pleasing esthetic outcome is equally important for patient satisfaction. Material selection has evolved from a purely technical decision to a critical factor influencing the functional, esthetic, and long-term success of restorations. Ceramics and polymethyl methacrylate (PMMA) are commonly used for the esthetic components, but space limitations often restrict the thickness of these materials, thereby affecting the final esthetic result.

For instance, the optimal thickness for zirconia abutments has been suggested to be around 0.9 mm to meet esthetic acceptability standards [10]. Millable lithium disilicate ceramics provide a gradient in translucency from incisal to cervical areas, influenced by crystalline content [11]. However, increasing the ceramic layer's thickness also increases its opacity increases [12], reducing the impact of the underlying substrate color and potentially compromising translucency, which is critical for achieving desirable esthetic outcomes [13].

Determining the optimal thickness of esthetic materials that minimally affects the final color, along with the ideal combination of substrate and esthetic material, remains an important area of study. Although many studies have evaluated these prostheses [2,3], few have specifically addressed the influence of the substrate and esthetic components on the final outcome of implant-supported restorations [2].

The purpose of this in vitro study was to evaluate the effect of the framework material (substrate), the esthetic material, and its thickness on the final color of the restoration. A secondary aim was to assess the influence of the cement on the final color reproduction. The null hypothesis was that the type of framework material (substrate), the type of esthetic material, and its thickness would not influence the final color of the restoration.

## Materials and methods

Six standard tessellation language (STL) files were generated using Fusion 360 software (Autodesk, San Rafael, California, United States) for this study. One file represented a substrate abutment disk (10 mm diameter, 8 mm thickness), while the remaining five files represented esthetic material disks (10 mm diameter) with thicknesses of 0.5, 1, 1.5, 2, and 2.5 mm. These files were then sent to a CAD-CAM milling center for test sample fabrication. The substrate disk was milled four times using different materials: hybrid PEEK-based ceramic-reinforced polymer (BioHPP, Bredent, Senden, Germany), chromium-cobalt alloy (CrCo, Duceralloy; Dentsply Sirona, Hanau, Germany), grade 5 titanium (Ti, Starbond Ti5 Disc, S & S Scheftner, Mainz, Germany), and white zirconium oxide ceramic (WZirCAD, Katana, Kuraray Noritake Dental, Tokyo, Japan). The A2 color esthetic material samples were milled from three materials, lithium disilicate ceramic (e.max CAD, Ivoclar Vivadent, Schaan, Liechtenstein), PMMA (Telio-CAD, Ivoclar Vivadent, Schaan, Liechtenstein), and zirconia oxide ceramic (e.max ZirCAD, Ivoclar Vivadent, Schaan, Liechtenstein), at five thickness levels (n=15).

Postprocessing included crystallizing and cooling the WZirCAD cylinder in a vacuum oven (inFire HTC speed; Dentsply Sirona, Charlotte, North Carolina, United States), with adjustments made for sintering shrinkage. Specimens were cleaned ultrasonically (Quantrex 210; L&R Manufacturing, Kearny, New Jersey, United States) and dried with oil-free compressed air. Metal substrates were polished with 2000 grit sandpaper, followed by felt and diamond solution polishing (Extec I; Extec, Enfield, Connecticut, United States), and then cleaned in deionized water for 10 minutes [14]. Esthetic samples underwent surface treatment with silicon carbide paper (600-1200 grit) using a processing machine (Buehler Phoenix Beta; Buehler, Lake Bluff, Illinois, United States) at 100 rpm for 15 seconds under cooling water, followed by crystallization as per the manufacturer's guidelines (Programat 300; Ivoclar Vivadent, Schaan, Liechtenstein). Ceramic specimens were left unglazed to maintain consistent thickness. The thickness of all esthetic samples was verified postprocessing using a digital micrometer (Digimatic Subler; Mitutoyo, Kawasaki, Japan).

Study design

**Figure 1:**
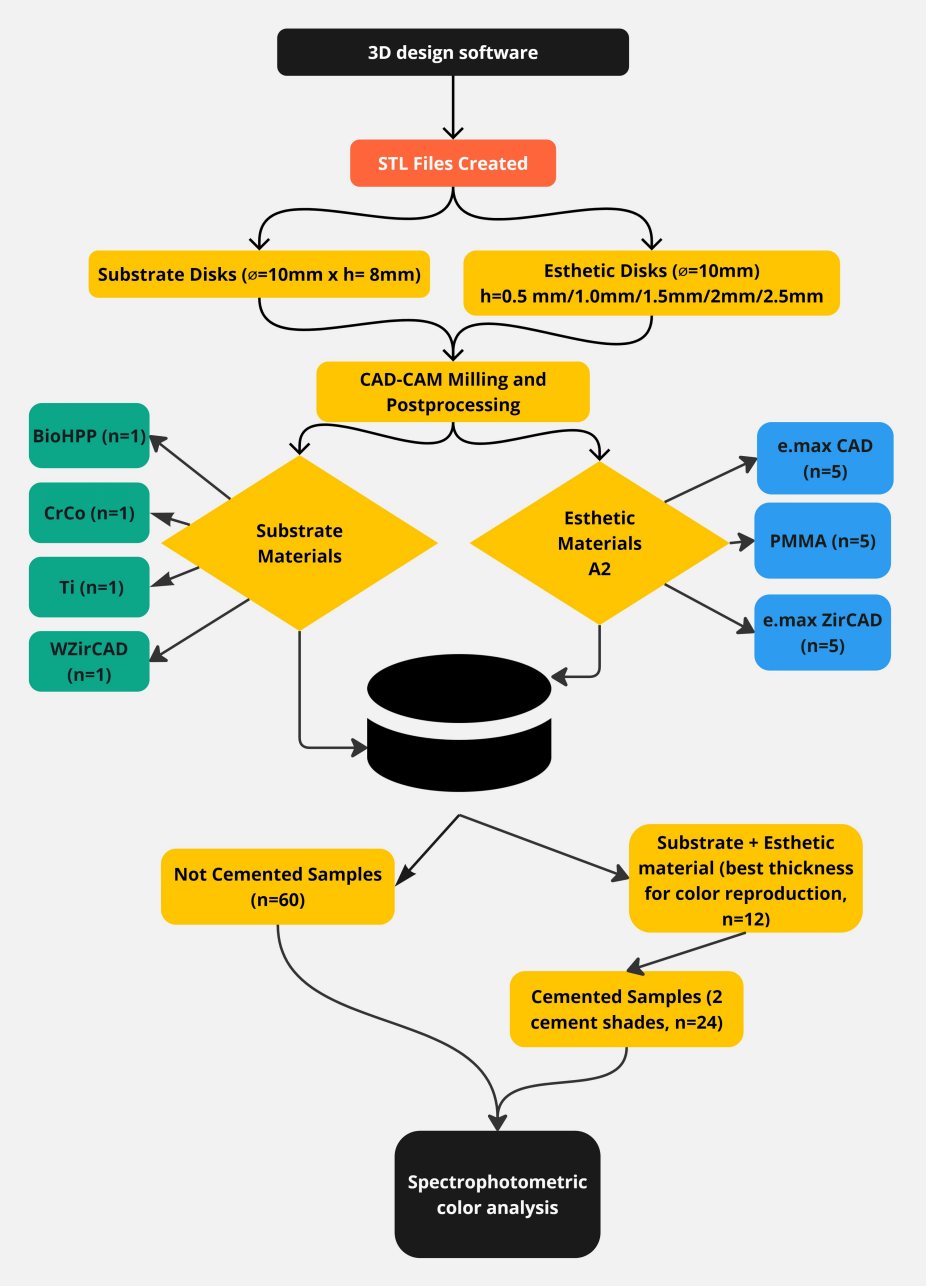
Study design e.max CAD: lithium disilicate ceramic; PMMA: polymethyl methacrylate; e.max ZirCAD: zirconia oxide ceramic; BioHPP: hybrid PEEK-based ceramic-reinforced high-performance polymer; CrCo: chromium-cobalt; Ti: grade 5 titanium; WZirCAD: white zirconium oxide ceramics

The sample size was calculated using mean (SD) from a previous study [15] in G*Power (Version 3.1.9.7, Heinrich-Heine-Universität Düsseldorf, Düsseldorf, Germany), yielding an estimated sample size of 3.01, making three determinations per group appropriate. Each esthetic material sample of varying thickness was tested on four substrate types (n=60), both without cement and with two cement shades (n=24) (Figure 1). Two examiners conducted three measurements per group, with a one-week interval, using the mean value. This resulted in 160 observations without cement and 72 with cement per examiner, totaling 464 observations.

Spectrophotometer calibration and measuring protocol

A VITA Easyshade® V spectrophotometer (Die VITA Zahnfabrik H. Rauter; Essen, Germany) was calibrated according to the manufacturer's instructions for color determinations, using the CIE Lab* color space (Commission Internationale de l'Éclairage) [16]. Two calibrated examiners performed measurements to ensure accuracy and reliability, with repeated testing validating the device's accuracy (kappa index >0.91 for repeatability and >0.89 for reliability) [17]. The spectrophotometer was aligned with the disk's diameter to prevent errors, and measurements were taken with and without cement. For non-cemented samples, optical fluid glycerin (Cargille Optical Gel) was applied to prevent light scattering [18]. For cemented samples, two shades of Variolink Esthetic try-in cement (neutral and white opaque, Ivoclar Vivadent, Schaan, Liechtenstein) were used, with a 250 g load applied for five seconds [19]. Measurements were conducted on a white background under standard lighting conditions.

The following color parameters were recorded relative to the A2 shade guide: ΔL* (lightness), ΔC* (chroma), ΔH* (hue), Δa* (red/green axis), Δb* (yellow/blue axis), and ΔE* (overall color difference). Color differences were calculated using the CIE Lab* system with the Euclidean formula: ΔE*=sqrt((ΔL*)²+(Δa*)²+(Δb*)²) [20].

Based on spectrophotometer calibration, these parameters categorized color match quality as good, fair, or in need of adjustment.

Parameters evaluated

Influence of Substrate, Esthetic Material Type, and Thickness on Clinical Perceptibility and Color Parameters

The study analyzed differences in ΔE*, ΔL*, ΔC*, and ΔH across tests using the same substrate and esthetic material but varying thicknesses, without the application of cement.

Influence of Cement on Final Crown Color

The impact of two different try-in cements on the color of the esthetic material-substrate assembly was assessed. To identify any significant variations, comparisons were made between substrate-thickness combinations that initially received a "good" rating (Figure 1).

Statistical analysis

Data were compiled in Excel (Microsoft Corporation, Redmond, Washington, United States) and analyzed using the nonparametric Kruskal-Wallis test to assess the effect of esthetic material thickness on various substrates. Post hoc pairwise comparisons were conducted with the Mann-Whitney U test and Bonferroni correction (p=0.05), using IBM SPSS Statistics for Windows, Version 20.0 (Released 2011; IBM Corp., Armonk, New York, United States).

## Results

The degree of concordance between the sets of measurements made by the two examiners was calculated as 0.994 with a 95% confidence interval of (0.991, 0.996).

Influence of substrate, esthetic material type, and thickness on clinical perceptibility and color parameters

The mean values and standard deviations of three measurements for ∆E*, ∆L*, ∆C*, and ∆H* taken by two examiners, categorized by substrate and esthetic material thickness, are presented in Tables 1-4. The standard deviation is less than 1 in all cases, indicating consistent measurements. 

**Table 1:** ∆E mean values (±SD) of the three measurements made by the two examiners, Kruskal-Wallis nonparametric test (α=0.05) by substrate and esthetic material e.max CAD: lithium disilicate ceramic; PMMA: polymethyl methacrylate; e.max ZirCAD: zirconia oxide ceramic; BioHPP: hybrid PEEK-based ceramic-reinforced high-performance polymer; CrCo: chromium-cobalt; Ti: grade 5 titanium; WZirCAD: white zirconium oxide ceramics

∆E* mean values (±SD)/substrate	Thickness (mm)	e.max CAD	PMMA	e.max ZirCAD	P-value
BioHPP	0.5	3.65±0.07	6.60±0.00	5.65±0.21	0.095
	1	0.60±0.00	7.55±0.07	3.45±0.07	0.095
	1.5	0.25±0.07	7.60±0.14	3.50±0.14	0.102
	2	2.55±0.07	3.45±0.21	2.4±00.00	0.095
	2.5	3.15±0.07	3.05±0.07	3.45±0.50	0.323
CrCo	0.5	5.35±0.21	6.75±0.07	6.75±0.21	1.000
	1	1.85±0.07	6.05±0.35	5.30±0.28	0.102
	1.5	1.55±0.07	7.25±0.07	3.45±0.64	0.102
	2	2.70±0.14	3.65±0.07	2.55±0.07	0.123
	2.5	3.00±0.00	3.95±0.21	2.75±0.07	0.095
Ti	0.5	7.65±0.07	3.60±0.00	7.75±0.21	0.156
	1	4.15±0.07	5.65±0.21	4.05±0.35	0.180
	1.5	3.05±0.07	5.90±0.14	3.50±0.1414	0.102
	2	4.15±0.07	1.55±0.07	3.60±0.0000	0.095
	2.5	3.75±0.07	3.55±0.07	3.20±0.0000	0.095
WZirCAD	0.5	7.00±0.00	6.75±0.07	8.25±0.0707	0.095
	1	2.75±0.07	7.30±0.00	4.35±0.0707	0.095
	1.5	1.75±0.07	7.65±0.07	3.50±0.1414	0.102
	2	2.75±0.07	2.95±0.07	2.65±0.0707	0.123
	2.5	3.20±0.00	4.05±0.07	2.95±0.0707	0.095

**Table 2:** ∆L mean values (±SD) of the three measurements made by the two examiners, Kruskal-Wallis nonparametric test (α=0.05) by substrate and esthetic material e.max CAD: lithium disilicate ceramic; PMMA: polymethyl methacrylate; e.max ZirCAD: zirconia oxide ceramic; BioHPP: hybrid PEEK-based ceramic-reinforced high-performance polymer; CrCo: chromium-cobalt; Ti: grade 5 titanium; WZirCAD: white zirconium oxide ceramics

∆L mean values (±SD)/substrate	Thickness (mm)	e.max CAD	PMMA	e.max ZirCAD	P-value
BioHPP	0.5	-0.30±0.28	4.25±0.21	3.00±0.28	0.102
	1	0.60±0.00	1.60±0.28	3.05±0.07	0.095
	1.5	-0.10±0.00	1.60±0.14	2.45±0.07	0.095
	2	-2.20±0.14	-0.65±0.07	-0.15±0.21	0.102
	2.5	-2.60±0.28	-0.50±0.14	0.25±0.35	0.102
CrCo	0.5	-	4.25±0.35	3.40±0.42	0.121
	1	0.35±0.21	2.40±0.14	4.15±0.07	0.102
	1.5	-0.10±0.00	2.00±0.14	2.65±0.50	0.095
	2	-1.55±0.07	-0.50±0.00	-0.05±0.64	0.123
	2.5	-2.50±0.14	-0.80±0.14	-0.25±0.07	0.102
Ti	0.5	-4.25±0.07	1.25±0.35	0.00±0.28	0.102
	1	-2.55±0.07	0.45±0.07	1.10±0.42	0.102
	1.5	-1.00±0.14	1.90±0.00	2.00±0.14	0.123
	2	-3.55±0.21	0.25±0.07	-1.20±0.28	0.102
	2.5	-3.20±0.28	-0.50±0.00	-1.20±0.00	0.089
WZirCAD	0.5	3.10±0.00	4.55±0.21	4.95±0.07	0.095
	1	1.10±0.14	1.40±0.42	3.50±0.00	0.148
	1.5	0.00±0.14	1.75±0.07	2.60±0.14	0.102
	2	-1.80±0.14	-0.35±0.07	0.20±0.14	0.102
	2.5	-2.70±0.14	-1.50±0.14	-0.10±0.00	0.095

**Table 3:** ∆C* mean values (±SD) of the three measurements made by the two examiners, Kruskal-Wallis nonparametric test (α=0.05) by substrate and esthetic material e.max CAD: lithium disilicate ceramic; PMMA: polymethyl methacrylate; e.max ZirCAD: zirconia oxide ceramic; BioHPP: hybrid PEEK-based ceramic-reinforced high-performance polymer; CrCo: chromium-cobalt; Ti: grade 5 titanium; WZirCAD: white zirconium oxide ceramics

∆C mean values (±SD)/substrate	Thickness (mm)	e.max CAD	PMMA	e.max ZirCAD	P-value
BioHPP	0.5	-3.60±0.14	5.20±0.42	-4.35±0.07	0.102
	1	-0.05±0.07	7.35±0.07	-1.40±0.00	0.095
	1.5	-0.20±0.00	7.40±0.14	-2.30±0.14	0.095
	2	-0.95±0.07	3.35±0.21	-2.20±0.00	0.095
	2.5	-1.50±0.42	3.05±0.07	-3.20±0.42	0.102
CrCo	0.5	-5.1±0.28	5.15±0.50	-5.20±0.14	0.121
	1	-1.80±0.00	5.60±0.42	-2.50±1.13	0.171
	1.5	-1.20±0.14	7.00±0.00	-1.95±0.35	0.095
	2	-2.10±0.14	3.60±0.00	-2.30±0.00	0.089
	2.5	-1.65±0.21	3.90±0.14	-2.55±0.07	0.102
Ti	0.5	-6.25±0.21	3.20±0.14	-7.15±0.21	0.102
	1	-3.30±0.00	5.65±0.21	-3.60±0.28	0.095
	1.5	-2.85±0.07	5.55±0.21	-2.50±0.14	0.102
	2	-2.20±0.00	1.15±0.07	-3.10±0.14	0.095
	2.5	-1.95±0.35	3.55±0.07	-2.55±0.21	0.102
WZirCAD	0.5	-6.10±0.00	4.70±0.42	-5.95±0.07	0.095
	1	-2.55±0.07	7.10±0.00	-2.15±0.07	0.095
	1.5	-1.70±0.14	7.45±0.07	-1.95±0.07	0.102
	2	-2.00±0.00	2.90±0.00	-2.35±0.07	0.089
	2.5	-1.65±0.21	3.75±0.07	-2.65±0.07	0.102

**Table 4:** ∆H* mean values (±SD) of the three measurements made by the two examiners, Kruskal-Wallis nonparametric test (α=0.05) by substrate and esthetic material e.max CAD: lithium disilicate ceramic; PMMA: polymethyl methacrylate; e.max ZirCAD: zirconia oxide ceramic; BioHPP: hybrid PEEK-based ceramic-reinforced high-performance polymer; CrCo: chromium-cobalt; Ti: grade 5 titanium; WZirCAD: white zirconium oxide ceramics

∆H mean values (±SD)/substrate	Thickness (mm)	e.max CAD	PMMA	e.max ZirCAD	P-value
BioHPP	0.5	1.15±0.07	0.95±0.21	6.80±0.14	0.123
	1	0.10±0.00	-1.20±0.14	2.20±0.00	0.089
	1.5	-0.30±0.00	-1.25±0.07	3.30±0.14	0.095
	2	-3.05±0.07	0.35±0.07	2.70±0.00	0.095
	2.5	-2.40±0.28	0.50±0.14	4.20±0.42	0.102
CrCo	0.5	4.25±0.07	2.30±0.42	8.70±0.57	0.121
	1	-1.15±0.07	-0.10±0.28	4.40±0.42	0.102
	1.5	-2.35±0.07	-0.90±0.00	3.40±0.42	0.095
	2	-1.75±0.07	0.55±0.07	3.35±0.07	0.102
	2.5	-2.10±0.14	0.20±0.14	3.70±0.14	0.102
Ti	0.5	4.55±0.35	3.30±0.14	11.25±0.35	0.102
	1	-0.25±0.07	0.60±0.14	4.90±0.42	0.102
	1.5	-1.30±0.00	-0.20±0.00	0.10±5.94	0.545
	2	-1.30±0.14	2.80±0.14	4.45±0.21	0.102
	2.5	-1.60±0.28	0.70±0.00	3.90±0.00	0.089
WZirCAD	0.5	4.65±0.07	3.85±0.21	10.90±0.14	0.102
	1	-0.05±0.07	0.25±0.07	4.85±0.07	0.102
	1.5	-1.45±0.07	-1.00±0.00	3.95±0.07	0.095
	2	-1.25±0.07	1.10±0.14	4.05±0.07	0.102
	2.5	-1.90±0.14	-0.05±0.07	4.05±0.07	0.102

ΔE* parameter: difference in color perception

The Kruskal-Wallis test showed no statistically significant differences in ∆E* values across groups of different substrates and esthetic materials (p>0.05), indicating that the type of esthetic material does not significantly affect final color perception (Table 1). Pairwise comparisons with Bonferroni correction also revealed no significant differences in ∆E* values.

**Figure 2:**
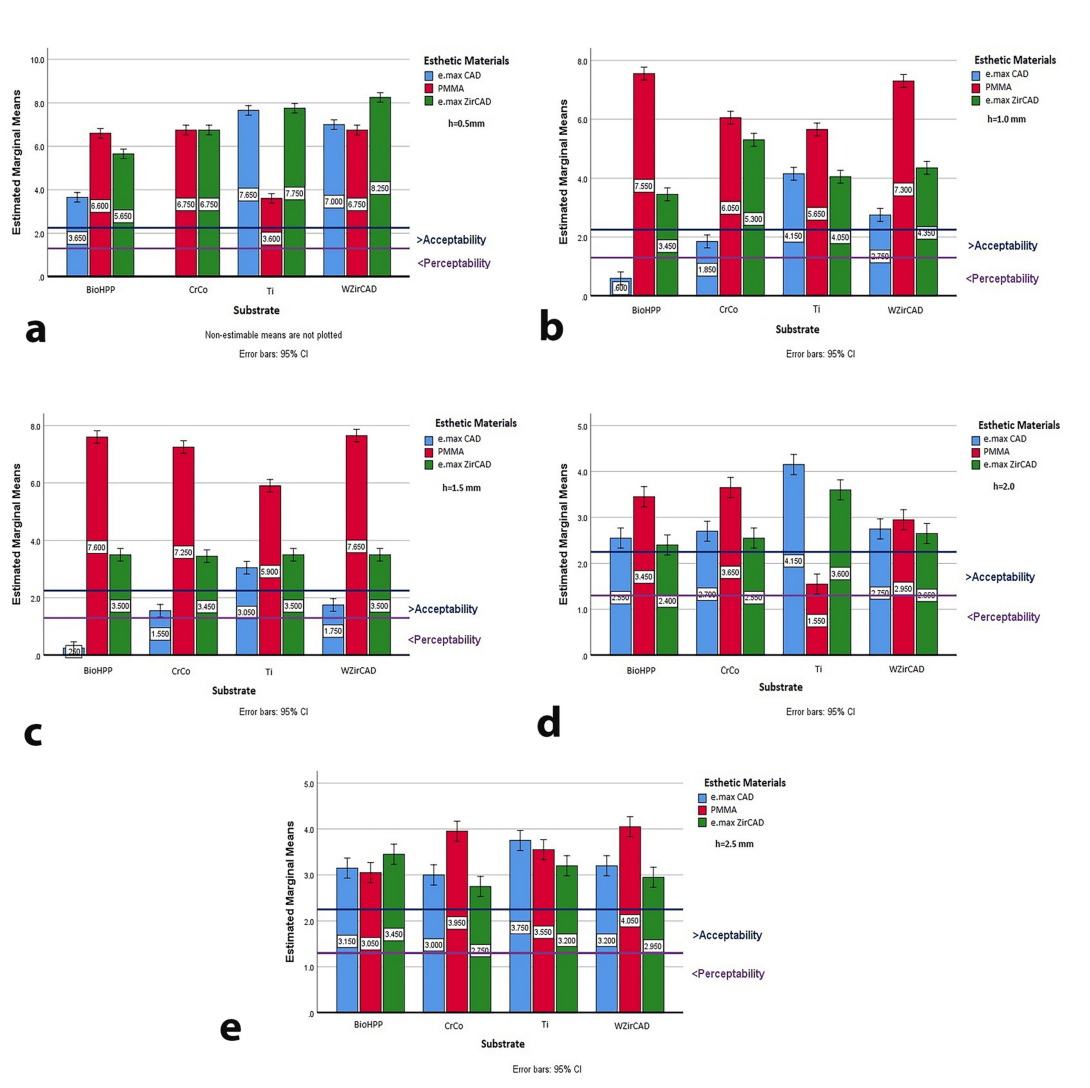
Estimated marginal means of ΔE* for different thicknesses of esthetic materials on all tested substrates (a) 0.5 mm esthetic material thickness; (b) 1 mm esthetic material thickness; (c) 1.5 mm esthetic material thickness; (d) 2 mm esthetic material thickness; (e) 2.5 mm esthetic material thickness e.max CAD: lithium disilicate ceramic; PMMA: polymethyl methacrylate; e.max ZirCAD: zirconia oxide ceramic; BioHPP: hybrid PEEK-based ceramic-reinforced high-performance polymer; CrCo: chromium-cobalt; Ti: grade 5 titanium; WZirCAD: white zirconium oxide ceramics

However, when assessing the influence of esthetic material thickness on ∆E*, p-values for all three materials were less than 0.05 (e.max CAD, p=0.0256; PMMA, p=0.0085; e.max ZirCAD, p=0.0055), indicating that thickness significantly impacts overall color difference. Analysis of mean ∆E* values by thickness (Figure 2a-2e) showed that at 0.5 mm and 2.5 mm, all values exceeded the 2.25 perception threshold. At 0.5 mm, the best results were achieved with BioHPP and e.max CAD (mean ∆E*=3.65) and with Ti and PMMA (mean ∆E*=3.60). At 1 mm, e.max CAD generally had the lowest ∆E* values, particularly with BioHPP (mean ∆E*=0.60) and CrCo (mean ∆E*=1.85). At 1.5 mm, e.max CAD continued to show the best results, with e.max ZirCAD maintaining a mean ∆E* of 3.5 across substrates. At 2 mm, the best result was PMMA on Ti (mean ∆E*=1.56), while at 2.5 mm, mean ∆E* values remained below 3.75, except for PMMA on CrCo and WZirCAD (Figure 2e). 

ΔL* parameter: difference in lightness (value)

The Kruskal-Wallis test revealed no statistically significant differences in ∆L* values across different substrates and esthetic materials (p>0.05), indicating that esthetic material type does not significantly affect lightness (Table 2). Pairwise comparisons with Bonferroni correction also showed no significant differences in ∆L* values.

When examining the effect of thickness on ∆L* values, both PMMA (p=0.005) and e.max ZirCAD (p=0.010) showed significant differences across thicknesses, suggesting that thickness significantly influences lightness for these materials. In contrast, e.max CAD did not show significant differences in ∆L* across thicknesses (p=0.080), indicating thickness has a minimal impact on its lightness.

**Figure 3:**
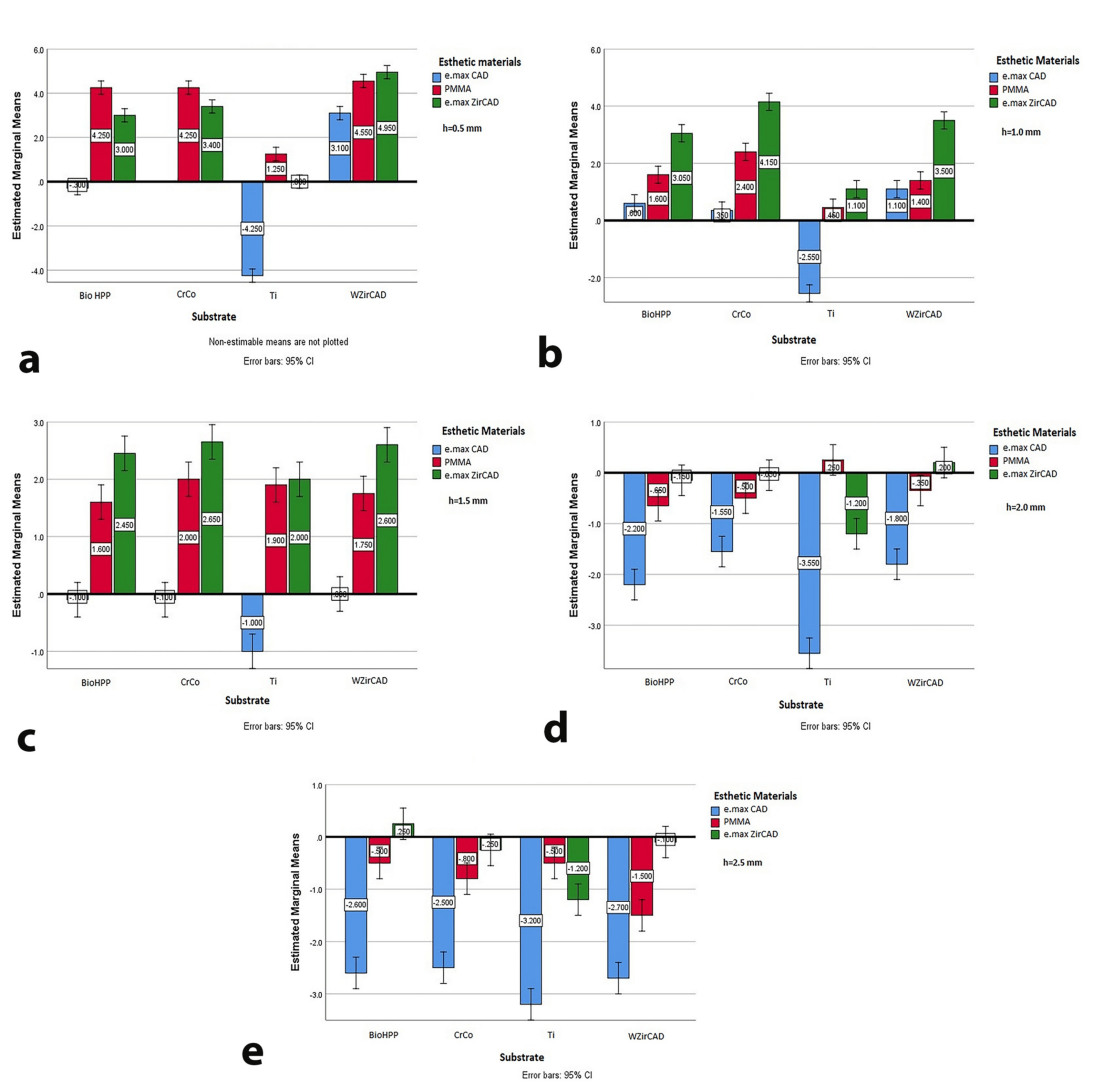
Estimated marginal means of ΔL* for different thicknesses of esthetic materials on all tested substrates (a) 0.5 mm esthetic material thickness; (b) 1 mm esthetic material thickness; (c) 1.5 mm esthetic material thickness; (d) 2 mm esthetic material thickness; (e) 2.5 mm esthetic material thickness e.max CAD: lithium disilicate ceramic; PMMA: polymethyl methacrylate; e.max ZirCAD: zirconia oxide ceramic; BioHPP: hybrid PEEK-based ceramic-reinforced high-performance polymer; CrCo: chromium-cobalt; Ti: grade 2 titanium; WZirCAD: white zirconium oxide ceramics

Analysis of mean ∆L* values by thickness showed a decrease in lightness when e.max CAD was applied to titanium, with the greatest reduction (mean ∆L*=-4.25) at 0.5 mm thickness. At 2 and 2.5 mm, lightness generally decreased across all substrates except e.max ZirCAD (Figure 3a-3e). Overall, increasing thickness tends to decrease ∆L* values, with more pronounced effects seen in e.max CAD and titanium substrates. Titanium generally exhibited more negative ∆L* values, particularly at greater thicknesses, compared to other substrates. e.max CAD and PMMA showed more significant changes in ∆L* with thickness, while e.max ZirCAD remained relatively stable across thickness levels. 

ΔC* parameter: difference in color intensity (chroma)

**Figure 4:**
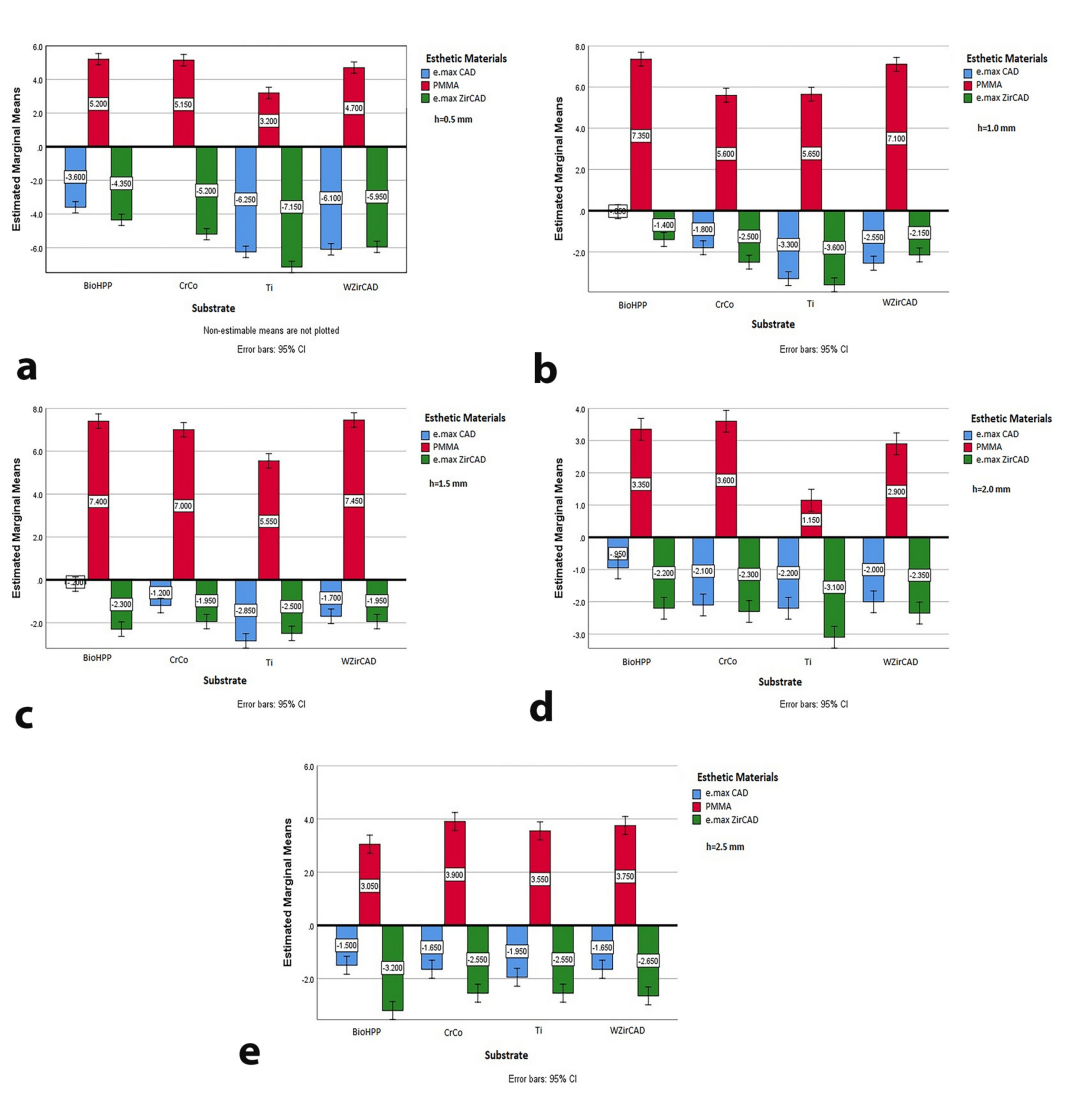
Estimated marginal means of ΔC* for different thicknesses of esthetic materials on all tested substrates (a) 0.5 mm esthetic material thickness; (b) 1 mm esthetic material thickness; (c) 1.5 mm esthetic material thickness; (d) 2 mm esthetic material thickness; (e) 2.5 mm esthetic material thickness e.max CAD: lithium disilicate ceramic; PMMA: polymethyl methacrylate; e.max ZirCAD: zirconia oxide ceramic; BioHPP: hybrid PEEK-based ceramic-reinforced high-performance polymer; CrCo: chromium-cobalt; Ti: grade 5 titanium; WZirCAD: white zirconium oxide ceramics

While substrate type does not significantly influence chroma (Table 3), esthetic material thickness does. Each material (e.max CAD, p=0.040; PMMA, p=0.004; e.max ZirCAD, p=0.014) shows significant chroma variation with thickness changes. PMMA consistently exhibited positive ΔC* values across substrates, indicating enhanced color intensity. In contrast, other substrate-material combinations generally showed a reduction in chroma, with titanium leading to the most significant decrease, particularly with e.max CAD and e.max ZirCAD. Except for PMMA, smaller ΔC* values were observed across all combinations at 1 mm and 1.5 mm thicknesses (Figure 4b, 4c). 

ΔH* parameter: difference in color saturation (hue)

**Figure 5:**
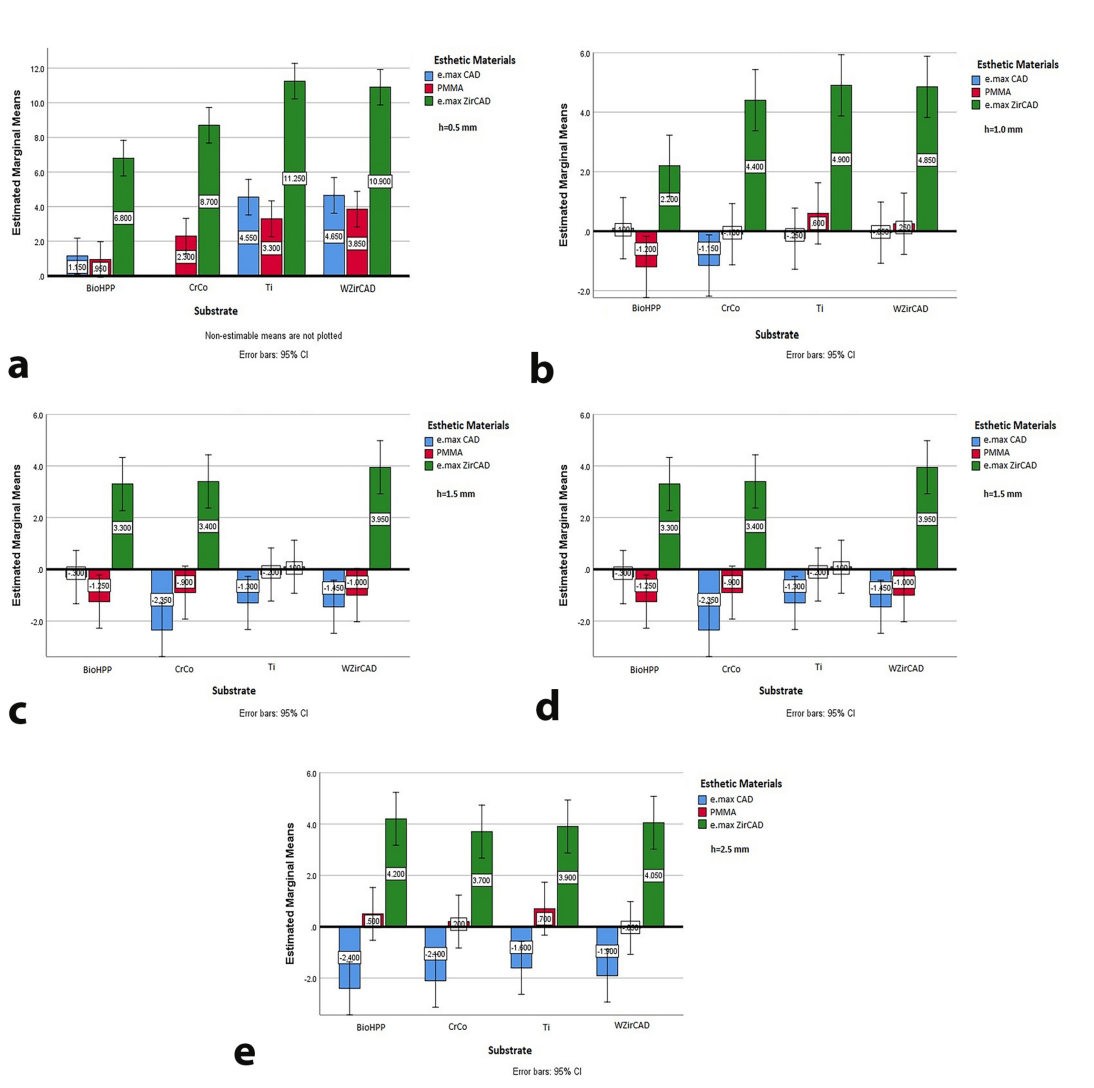
Estimated marginal means of ΔH* for different thicknesses of esthetic materials on all tested substrates (a) 0.5 mm esthetic material thickness; (b) 1 mm esthetic material thickness; (c) 1.5 mm esthetic material thickness; (d) 2 mm esthetic material thickness; (e) 2.5 mm esthetic material thickness e.max CAD: lithium disilicate ceramic; PMMA: polymethyl methacrylate; e.max ZirCAD: zirconia oxide ceramic; BioHPP: hybrid PEEK-based ceramic-reinforced high-performance polymer; CrCo: chromium-cobalt; Ti: grade 5 titanium; WZirCAD: white zirconium oxide ceramics

There is no statistically significant difference in ∆H* across different substrates, indicating that substrate type does not significantly impact the hue of esthetic materials (Table 4). However, the thickness of esthetic materials, particularly PMMA (p=0.006) and e.max ZirCAD (p=0.020), significantly affects hue. In contrast, e.max CAD shows no significant thickness-related influence on color saturation (p=0.084) and generally exhibits negative ΔH* values with increasing thickness, indicating decreased color saturation (Figure 5a-5e). Exceptions include Ti and WZirCAD substrates, where positive ΔH* values are observed at lower thicknesses (0.5 mm). PMMA displays both positive and negative ΔH* values depending on the substrate, but with less variation compared to e.max ZirCAD. e.max ZirCAD shows significant positive ΔH* values at lower thicknesses (0.5 and 1 mm) and on specific substrates (e.g., CrCo, Ti, WZirCAD), indicating higher color saturation compared to the other two materials (Figure 5a-5b). 

Influence of cement on final crown color

The influence of cement was assessed using esthetic material thicknesses closest to the A2 color determined earlier: 1.5 mm for e.max CAD, 2 mm for PMMA, and 2.5 mm for e.max ZirCAD. The Kruskal-Wallis test with Bonferroni correction revealed no significant differences among the neutral, opaque, and no cement groups. Overall, cement application did not significantly affect final color parameters (ΔE*) across different esthetic materials (Table 5).

**Table 5:** ∆E: Kruskal-Wallis nonparametric test for the influence of two shades of cement on the final color of the restoration e.max CAD: lithium disilicate ceramic; PMMA: polymethyl methacrylate; e.max ZirCAD: zirconia oxide ceramic; BioHPP: hybrid PEEK-based ceramic-reinforced high-performance polymer; CrCo: chromium-cobalt; Ti: grade 5 titanium; WZirCAD: white zirconium oxide ceramics

∆E*/substrate	Esthetic material (thickness)	Neutral cement	Opaque cement	No cement	P-value
BioHPP	e.max CAD (1.5 mm)	2.10±0.28	2.60±0.00	0.25±0.07	0.53
	PMMA (2 mm)	3.50±0.14	3.75±0.07	3.45±0.21	0.11
	e.max ZirCAD (2.5 mm)	3.35±0.21	3.45±0.21	3.45±0.50	0.92
CrCo	e.max CAD (1.5 mm)	3.20±0.00	4.35±0.07	1.55±0.07	0.53
	PMMA (2 mm)	3.00±0.28	4.95±0.07	3.65±0.07	0.56
	e.max ZirCAD (2.5 mm)	4.00±0.28	3.75±0.07	2.75±0.07	0.05
Ti	e.max CAD (1.5 mm)	4.05±0.07	6.10±0.28	3.05±0.07	0.56
	PMMA (2 mm)	3.20±0.14	4.30±0.01	1.55±0.07	0.56
	e.max ZirCAD (2.5 mm)	4.05±0.07	4.30±0.00	3.20±0.00	0.38
WZirCAD	e.max CAD (1.5 mm)	2.30±0.00	2.85±0.07	1.75±0.07	0.53
	PMMA (2 mm)	2.80±0.14	3.70±0.14	2.95±0.07	0.07
	e.max ZirCAD (2.5 mm)	3.60±0.14	3.65±0.21	2.95±0.07	0.06

However, near-significant effects were noted for e.max ZirCAD on CrCo (p=0.05) regarding color perception, as well as for color intensity in e.max ZirCAD on CrCo and e.max CAD on WZirCAD, and for lightness in PMMA on Ti and e.max ZirCAD on WZirCAD (all p=0.05). Cement application did not affect saturation in any tests (Tables 6-8). 

**Table 6:** Kruskal-Wallis nonparametric test for the influence of two shades of cement on ∆L* e.max CAD: lithium disilicate ceramic; PMMA: polymethyl methacrylate; e.max ZirCAD: zirconia oxide ceramic; BioHPP: hybrid PEEK-based ceramic-reinforced high-performance polymer; CrCo: chromium-cobalt; Ti: grade 5 titanium; WZirCAD: white zirconium oxide ceramics

∆L*/substrate	Esthetic material (thickness)	Neutral cement	Opaque cement	No cement	P-value
BioHPP	e.max CAD (1.5 mm)	-1.40±0.57	0.25±0.07	-0.10±0.00	0.398
	PMMA (2 mm)	-1.35±0.35	-1.50±0.14	-0.65±0.07	0.062
	e.max ZirCAD (2.5 mm)	-0.50±0.00	0.20±0.14	0.25±0.35	0.126
CrCo	e.max CAD (1.5 mm)	-1.85±0.07	-1.75±0.07	-0.10±0.00	0.450
	PMMA (2 mm)	-1.90±0.00	-2.90±0.00	-0.50±0.00	0.362
	e.max ZirCAD (2.5 mm)	-0.40±0.42	-0.40±0.00	-0.25±0.07	0.355
Ti	e.max CAD (1.5 mm)	-2.00±0.70	-3.90±0.42	-1.00±0.14	0.555
	PMMA (2 mm)	-2.40±0.14	-2.50±0.00	0.25±0.07	0.052
	e.max ZirCAD (2.5 mm)	-0.75±0.07	-0.45±0.07	-1.20±0.00	0.398
WZirCAD	e.max CAD (1.5 mm)	-0.75±0.07	-0.45±0.07	0.00±0.14	0.555
	PMMA (2 mm)	-0.95±0.21	-1.45±0.21	-0.35±0.07	0.555
	e.max ZirCAD (2.5 mm)	0.00±0.14	0.35±0.07	-0.10±0.00	0.0522

**Table 7:** Kruskal-Wallis nonparametric test for the influence of two shades of cement on ∆C* e.max CAD: lithium disilicate ceramic; PMMA: polymethyl methacrylate; e.max ZirCAD: zirconia oxide ceramic; BioHPP: hybrid PEEK-based ceramic-reinforced high-performance polymer; CrCo: chromium-cobalt; Ti: grade 5 titanium; WZirCAD: white zirconium oxide ceramics

∆C*/substrate	Esthetic material (thickness)	Neutral cement	Opaque cement	No cement	P-value
BioHPP	e.max CAD (1.5 mm)	-1.25±0.21	-2.55±0.07	-0.20±0.00	0.398
	PMMA (2 mm)	2.75±0.35	3.40±0.00	3.35±0.21	0.126
	e.max ZirCAD (2.5 mm)	-3.05±0.21	-3.20±0.28	-3.20±0.42	0.784
CrCo	e.max CAD (1.5 mm)	-2.60±0.00	-3.95±0.07	-1.20±0.14	0.535
	PMMA (2 mm)	2.15±0.35	3.90±0.14	3.60±0.00	0.398
	e.max ZirCAD (2.5 mm)	-3.65±0.35	-3.40±0.00	-2.55±0.07	0.052
Ti	e.max CAD (1.5 mm)	-3.45±0.50	-4.60±0.00	-2.85±0.07	0.535
	PMMA (2 mm)	1.70±0.00	3.30±0.14	1.15±0.070	0.535
	e.max ZirCAD (2.5 mm)	-3.55±0.07	-3.90±0.00	-2.55±0.2121	0.535
WZirCAD	e.max CAD (1.5 mm)	-2.15±0.07	-2.80±0.00	-1.70±0.14	0.535
	PMMA (2 mm)	2.55±0.07	3.35±0.070	2.90±0.00	0.398
	e.max ZirCAD (2.5 mm)	-3.25±0.21	-3.25±0.21	-2.65±0.07	0.060

**Table 8:** Kruskal-Wallis nonparametric test for the influence of two shades of cement on ∆H* e.max CAD: lithium disilicate ceramic; PMMA: polymethyl methacrylate; e.max ZirCAD: zirconia oxide ceramic; BioHPP: hybrid PEEK-based ceramic-reinforced high-performance polymer; CrCo: chromium-cobalt; Ti: grade 5 titanium; WZirCAD: white zirconium oxide ceramics

∆H*/substrate	Esthetic material (thickness)	Neutral cement	Opaque cement	No cement	P-value
BioHPP	e.max CAD (1.5 mm)	-2.65±0.21	-1.00±0.00	-0.30±0.00	0.380
	PMMA (2 mm)	0.65±0.21	0.40±0.00	0.35±0.07	0.0751
	e.max ZirCAD (2.5 mm)	4.00±0.28	4.45±0.21	4.20±0.42	0.255
CrCo	e.max CAD (1.5 mm)	-0.25±0.07	1.90±0.00	-2.35±0.07	0.535
	PMMA (2 mm)	2.80±0.28	1.75±0.07	0.55±0.07	0.555
	e.max ZirCAD (2.5 mm)	5.30±0.42	5.50±0.14	3.70±0.14	0.062
Ti	e.max CAD (1.5 mm)	1.05±0.50	2.80±0.14	-1.30±0.00	0.398
	PMMA (2 mm)	3.65±0.07	2.20±0.14	2.80±0.14	0.555
	e.max ZirCAD (2.5 mm)	5.65±0.07	6.15±0.07	3.90±0.00	0.398
WZirCAD	e.max CAD (1.5 mm)	-0.20±0.00	1.00±0.14	-1.45±0.07	0.535
	PMMA (2 mm)	2.15±0.07	1.25±0.21	1.10±0.14	0.100
	e.max ZirCAD (2.5 mm)	5.15±0.21	5.20±0.28	4.05±0.07	0.062

## Discussion

This study examines the impact of substrate material, esthetic material thickness, and cement on the final color reproduction in implant-supported fixed restorations. The findings highlight the intricate interaction of these factors in achieving optimal esthetic results, with significant implications for clinical practice. Notably, the literature lacks comprehensive data on the effects of substrate material and varying esthetic material thicknesses, particularly in hybrid prosthetic restorations on dental implants, where the balance between mechanical strength and esthetic quality is crucial.

The null hypothesis, positing that the framework material and esthetic material type would not affect the final restoration color, was partially accepted. However, the thickness of the esthetic material was found to significantly influence the overall color difference (ΔE*), with p-values <0.05 for all materials tested: e.max CAD, PMMA, and e.max ZirCAD. Each esthetic material exhibited a specific thickness at which color discrepancy reached clinical acceptability, with deviations from this thickness leading to visible color differences (Figure 2a-2e). The optimal esthetic results were observed at a 1 mm thickness, particularly with e.max CAD on BioHPP and CrCo substrates, where ΔE* values approached the clinical acceptability threshold.

The human eye's ability to perceive color differences varies, with ΔE* <1 considered undetectable, values between 1 and 3.3 visible to trained observers and clinically acceptable [21], and values >3.3 noticeable to the general public and thus clinically unacceptable [22]. This study employed a ΔE* range of 1.30 (perceptibility threshold (PT)) to 2.25 (acceptability threshold (AT)) as the standard for clinical perceptibility and acceptability. This range is consistent with ISO/TR 13028642:2016, which suggests a 50:50% acceptability and perceptibility evaluation. While other studies propose different thresholds [23,24], our study used a lower AT value (∆E*=2.25) compared to others. According to Paravina et al. [25,26], a PT of ≤1.2 and an AT of ≤2.7 categorize mismatches into three types: a (≤5.4, moderately unacceptable), b (≤8.1, clearly unacceptable), and c (>8.1, extremely unacceptable). In this study, all esthetic materials at 2 and 2.5 mm thicknesses fell into the clinically moderate unacceptable category, with only PMMA at 1.5 mm exceeding the type a limit. No esthetic materials at any thickness surpassed the 8.1 threshold for extreme unacceptability.

Contrary to Tabatabaian et al. [10], who recommended a 0.9 mm thickness for zirconia esthetic materials with an associated ∆E*=3.3, our study found that an e.max ZirCAD thickness of 1.5 mm produced consistent ∆E* values (3.5) across all substrates, although exceeding our chosen AT. For thinner esthetic materials, ∆E* values exceeded 5.30 for CrCo at 1 mm thickness and more than 5.8 on all substrates at 0.5 mm thickness. Thicker layers generally masked the underlying substrate color more effectively, reducing ∆E* values, consistent with other studies [27,28].

Specific material-substrate combinations revealed that for 0.5 mm and 2.5 mm thicknesses, all combinations exceeded the set color difference threshold, with BioHPP with e.max CAD and Ti with PMMA showing the best results at 0.5 mm (Figure 3a). PMMA generally exhibited higher ∆E* values compared to e.max CAD and e.max ZirCAD, suggesting its susceptibility to color changes or reduced effectiveness in masking the substrate, aligning with studies that highlight the lower color stability of PMMA [29].

Substrate material also influenced color reproduction. Ti substrates showed significant reductions in lightness (ΔL*) and chroma (ΔC*), particularly with e.max CAD and e.max ZirCAD, aligning with prior research indicating that Ti can impart a metallic hue [18,30]. BioHPP substrates generally produced better color matching, especially with e.max CAD.

Different cement shades (neutral and opaque) nearly significantly influenced color perception, particularly with e.max ZirCAD on CrCo substrates (p=0.05). Although overall differences were not statistically significant, the trend suggests that cement shade can subtly alter final color outcomes, crucial in esthetically demanding clinical scenarios. For PMMA and e.max ZirCAD, cement variations influenced lightness and chroma, albeit to a lesser extent.

The study underscores the importance of selecting appropriate esthetic materials and their thickness to achieve optimal esthetic outcomes in implant-supported restorations. While CAD-CAM technology ensures precision, material properties and their interactions with substrates and cements must be carefully considered. The study suggests a preference for BioHPP with moderate material thicknesses (around 1 mm) for enhanced esthetic outcomes.

Based on the study results, the following substrate-esthetic material combinations are recommended: For BioHPP, e.max CAD at 1 mm thickness enhances lightness and saturation with unaltered intensity; at 1.5 mm, it optimizes lightness and intensity with a slight saturation decrease. For Ti, PMMA at 2 mm thickness slightly increases lightness, intensity, and saturation. For CrCo, e.max CAD at 1 mm thickness slightly increases lightness with reduced intensity and saturation; at 1.5 mm, it reduces lightness, intensity, and saturation. For WZirCAD, no material met the optimal conditions, but e.max CAD was closest.

This study's limitations include its in vitro design, which, while providing controlled conditions, may not fully replicate in vivo complexities. Future research should explore long-term clinical outcomes and patient-reported satisfaction to validate these findings. Expanding the range of substrate and cement combinations could offer a more comprehensive understanding of their impact on esthetic outcomes.

Only samples scoring a "good" outcome were tested with the two cement shades, as accurate color reproduction is essential. In clinical practice, any color mismatches necessitate cosmetic adjustments before final cementation and use.

## Conclusions

Within the study's limitations, the following conclusions were drawn: Optimal esthetic results were achieved with a 1 mm thickness of esthetic materials, particularly e.max CAD on BioHPP and CrCo substrates. Both thinner (0.5 mm) and thicker (2.5 mm) materials often exceeded acceptable color thresholds. PMMA showed higher ΔE* values, indicating lower color stability and effectiveness in masking substrates compared to e.max CAD and e.max ZirCAD. Cement shade had a near-significant impact on color perception, especially with e.max ZirCAD on CrCo substrates, underscoring the importance of cement selection for optimal esthetics.

For enhanced outcomes, BioHPP with e.max CAD at 1 or 1.5 mm thickness is recommended, while Ti substrates pair best with 2 mm PMMA and CrCo substrates with e.max CAD at 1 or 1.5 mm thickness. Further in vivo studies are necessary to validate these findings, assess long-term clinical outcomes, and explore a wider range of substrate and cement combinations.
